# The complete mitochondrial genome of *Rhinogobius duospilus* (Gobiidae: Gobionellinae)

**DOI:** 10.1080/23802359.2020.1823279

**Published:** 2020-09-22

**Authors:** Hongyu Tan, Yanyan Yang, Man Zhang, Xiuli Chen

**Affiliations:** a Guangxi Colleges and Universities Key Laboratory of Aquatic Healthy Breeding and Nutrition Regulation, College of Animal Science and Technology, Guangxi University, Nanning, Guangxi, China; b School of Marine Sciences, Guangxi University, Nanning, Guangxi, China; c Guangxi Academy of Fishery Sciences, Nanning, Guangxi, China

**Keywords:** *Rhinogobius duospilus*, Gobionellinae, mitogenome, phylogenetic

## Abstract

*Rhinogobius duospilus* is a small freshwater fish with brilliant color in southern China, belonging to the subfamily Gobionellinae. In this study, the complete mitochondrial genome of 16,496 bp from *R. duospilus* was reported for the first time. It composed of 13 protein-coding genes, 22 tRNA genes, 2 rRNA genes, and 2 non-coding genes. Phylogenetic tree showed that *R. duospilus* formed a separate lineage. The findings here would be helpful to further researches of *R. duospilus.*

*Rhinogobius duospilus* is a small benthic freshwater fish in the subfamily Gobionellinae (Teleostei: Gobiidae) that is mainly distributed in southern China and classified as a new species since 1935 (Herre 1935, Li et al. 2008). Because of the unique spawning behavior and colorful appearance, it is well-liked by ornamental fish lovers. Previous studies of *R. duospilus* had focused on taxonomy and morphology, and the population structure based on genetic markers had been reported until recently (Feng et al. 2017; Li et al. 2008; Wu et al. 2016), but the characterization of the complete mitogenome remain unknown. Here, we reported the complete mitochondrial genome of *R. duospilus* for the first time, contributing to the phylogenetic and genetic protection researches of this species.

Specimens of *R. duospilus* were collected from Guilin City, Guangxi, China (25°16′25.00″N, 100°17′24.07″E) and were stored at −20 °C in Guangxi Colleges and Universities Key Laboratory of Aquatic Healthy Breeding and Nutrition Regulation, Guangxi University. The total genomic DNA was extracted from the dorsal muscle. The sequences were amplified by PCR with twenty pairs of primers and then the mitochondrial genome was annotated though MitoAnnotator (Iwasaki et al. 2013).

The complete mitochondrial genome of *R. duospilus* was 16,496 bp in length (GenBank accession no. MH127918), with A + T-biased (the G + C content was 47.3%). The overall base nucleotide composition was 27.1% A, 30.3% C, 17.0% G, 25.7% T. The mitochondrial genome contains 13 protein-coding genes (PCGs), 22 tRNA genes, 2 rRNA genes, 2 non-coding genes (light strand origin of replication (O_L_) and control region (D-loop)), which were high degree of conservation among *R. duospilus* and other Gobionellinae fishes. The 12S and 16S rRNA genes were 953 and 1684 bp in length, respectively. The O_L_ was 32 bp in length and located between *tRNA^Asn^* and *tRNA^Cys^*and the D-loop was 843 bp in length and located between *tRNA^Pro^* and *tRNA^Phe^.* Among the 13 PCG_S_, there were 12 genes with ATG as the start codon, except for *COI* with GTG as the start codon. In addition, six PCG_S_ had a complete TAA stop codon and two PCGs had a complete TAG stop codon, while five PCGs (*COII*, *COIII*, *ND3*, *ND4* and *CYTB*) showed an incomplete stop codon (T).

The maximum likelihood tree was constructed based on 13 mitochondrial PCGs of 11 Gobionellinae species using MEGA 7.0 software (Kumar et al. 2016). *Acanthogobius hasta* and *Lophiogobius ocellicauda* were included as outgroups. Evolutionary model was inferred by the program PhyML 3.0, where the substitution model GTR + G+I was considered as the most appropriate to the data (Guindon et al. 2010). Compared with the other Gobionellinae species, *R. duospilus* formed a separate lineage, which was showed in the phylogenetic tree (Figure 1). These results provided an important basis for further studies on mitochondrial genome and phylogenetics of *R. duospilus*.

**Figure 1.**
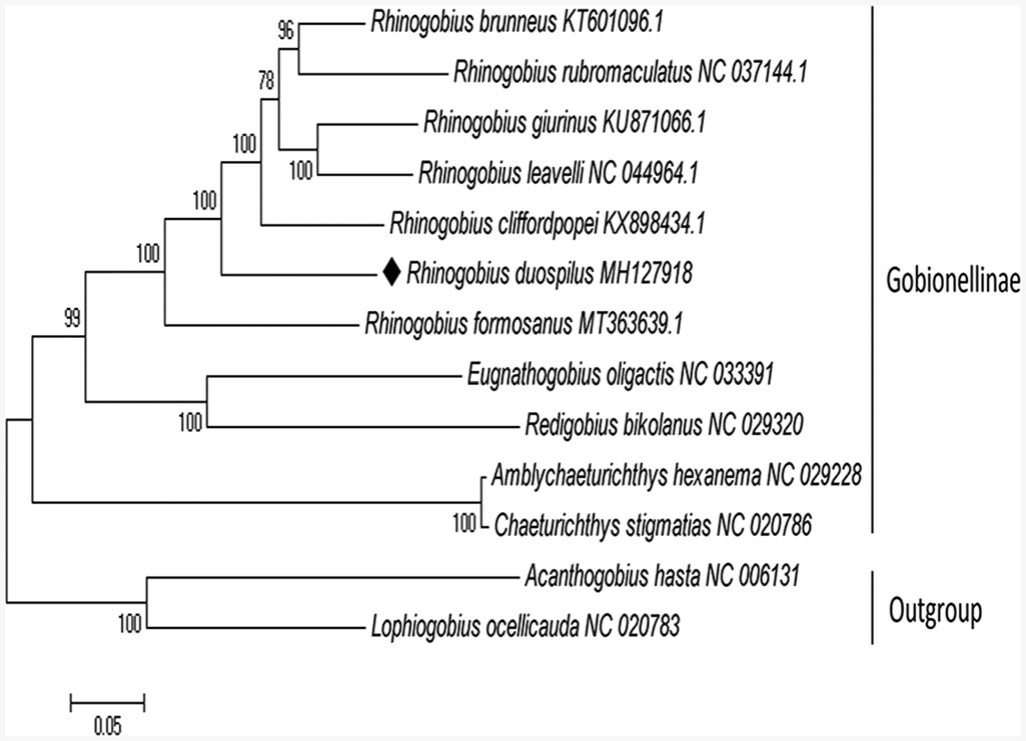
Phylogenetic tree of *Rhinogobius duospilus*, based on 13 concatenated mitochondrial PCGs from 11 Gobionellinae species, with *Acanthogobius hasta* and *Lophiogobius ocellicauda* as the outgroup. The mitogenomic information of *Rhinogobius duospilus* is marked with rhombus.

## Data Availability

The data that support the findings of this study are openly available in GenBank at https://www.ncbi.nlm.nih.gov/nuccore/MH127918, GenBank Accession Number: MH127918.
